# Dynamic covalent polymer films formed by structural metamorphosis at nanoparticle surfaces

**DOI:** 10.1039/d6py00112b

**Published:** 2026-04-20

**Authors:** Matthew J. Priestley, David A. Fulton

**Affiliations:** a Chemistry – School of Natural and Environmental Sciences, Newcastle, University Bedson Building Newcastle-upon-Tyne NE1 7RU UK david.fulton@ncl.ac.uk

## Abstract

Wrapping nanoscale objects within robust polymer films offers a powerful strategy to control surface properties, stability and functionality, yet achieving uniform, individually wrapped nanoparticles remains challenging. Here we report the first example of nanoparticle wrapping using dynamic covalent single-chain polymer nanoparticles (SCPNs) that undergo a concentration-triggered structural metamorphosis into an intermolecularly-crosslinked polymer film at the particle surface. Imidazole-decorated SCPNs bind multivalently to Ni-NTA-functionalised silica nanoparticles, where confinement and local concentration induce dynamic acylhydrazone exchange, transforming intramolecular crosslinks into intermolecular ones. Dynamic light scattering, zeta potential measurements and transmission electron microscopy confirm the formation of monodisperse, individually wrapped nanoparticles bearing thin, stable polymer shells that cannot be displaced by competitive ligands. In contrast, non-crosslinked imidazole-functionalised polymers are only able to form coatings that are largely removed. This strategy provides a versatile platform for creating nanoparticle wrappers and establishes a conceptual framework for extending polymer wrapping to complex and biologically relevant nanoscale objects.

## Abstract 2

We report a structural metamorphosis approach to wrap nanoparticles within cross-linked polymer films.

## Graphical abstract

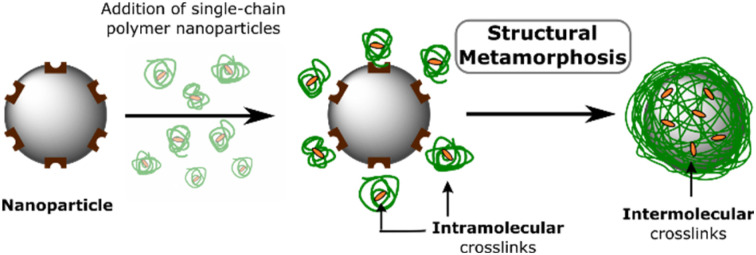

## Introduction

Polymer wrappers are ubiquitous in everyday life, where they provide durable protective barriers that shield objects from harsh external conditions, extend shelf life, and enable safe and convenient transport.^1,2^ Recent scientific advances have enabled the creation of nanoscale objects, such as engineered nanoparticles and virus-like particles (VLPs) that play increasingly important roles in technological and biomedical applications.^3–8^ Wrapping nanoscale objects in polymer films is therefore expected to offer advantages analogous to macroscopic wrapping, while additionally enabling the introduction of functionality and biocompatibility, control over surface charge, and enhanced colloidal stability of nanoparticles.

There are numerous established approaches to coating objects in polymer films such as electrostatic layer-by-layer methods^9^ and covalent grafting, where polymer chains are either grafted onto or grown from surfaces.^10,11^ While these strategies are effective for modifying particle properties, each has inherent limitations. Layer-by-layer coatings consist of discrete polymer chains held at the surface through electrostatic interactions and are therefore susceptible to removal by competitive ligands or environmental changes. In contrast, grafted polymer coatings are covalently attached to the particle surface and thus are non-displaceable; however, they also typically require additional chemical processes and careful consideration of surface chemistry, reaction conditions and compatibility with the underlying particle. An alternative strategy that combines the simplicity of non-covalent surface binding with the robustness of a covalently interconnected polymer film would therefore be highly attractive.

We have developed a new approach to wrapping particle surfaces which employs a structural metamorphosis of polymer architectures.^12,13^ This process can be defined as a transition between two discrete topographies on the supramolecular level, wherein an external stimulus/template triggers the reorganisation of polymer connectivity, rather than addition of further chemical reagents. Recent studies have applied metamorphological approaches utilizing dynamic covalent or non-covalent bonds to create stimuli responsive smart polymers and to modulate material and mechanical properties.^14–17^ While conventional polymer coatings comprise discrete chains anchored to a surface *via* intermolecular forces or covalent grafting, we define a polymer wrapper as a continuous, interconnected network that encapsulates the particle to provide a robust protective barrier. Central to our approach is a class of single-chain polymer nanoparticle (SCPN)^18^ ‘wrapping agents’ featuring internal dynamic covalent acylhydrazone crosslinks.^19–22^ At pH 4.5,^23,24^ the system is in a state of dynamic equilibria with dynamic bonds breaking and reforming continuously, until the most thermodynamically stable configuration is reached. Under dilute conditions, the entropic cost of crosslinking with another polymer chain is greater than that for a polymer crosslinking with itself. Therefore, these SCPNs maintain kinetic stability and exist as discrete, intramolecularly crosslinked entities. However, upon concentration, the entropic penalty of finding another polymer chain is removed thus allowing the release of strain and the unfolding of the SCPN *via* intermolecular component exchange to form an interconnected polymer network.

**Fig. 1:**
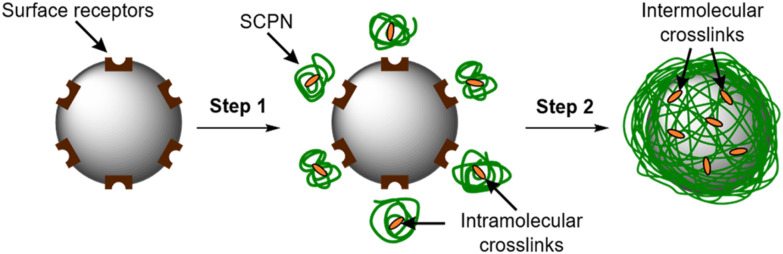
The structural metamorphosis wrapping approach. Step (1): Polymers bind to the particle. This binding is driven by interactions between polymers presenting a multivalent display of ligands and surface receptors. Step (2): On account of their high local concentration at the particle surface, the polymers are now spatially close to one another to trigger intra- to intermolecular crosslinker exchange to give a continuous polymer film around the particle.

When performed at a particle surface, this concentration yields a polymeric film that wraps the surface. Polymer confinement at the surface is facilitated by the interactions between receptors on the particle and a multivalent display of complementary ligands on the SCPNs (Fig. 1, step 1). Once enough polymer has adsorbed onto the particle surface, the local concentration of SCPNs is large enough to trigger the component exchange to occur (Fig. 1, step 2). The transition from intra- to intermolecular crosslinks constitutes the formation of a continuous polymer film with an interconnected nature providing structural integrity, maintaining the wrapper even under conditions whereby the initial surface-binding interactions have been disrupted.

Although we have successfully demonstrated this metamorphosis through the wrapping of 2D substrates^12^ and microparticles,^13^ its application to nanoscopic surfaces has remained elusive. Herein, we report the first instance of the wrapping of a nanoparticle within a polymer film, a significant advancement that will enhance the possibilities of nanoparticles within applications.

## Results and discussion

The formation of individually wrapped nanoparticles—where each particle is wrapped and there is minimal formation of multiparticle aggregates—is challenging. Nanoparticle surfaces are often chemically modified to introduce steric and/or electronic barriers that suppress aggregation, which otherwise occurs to reduce overall surface energy. The complexation of polymer chains upon the nanoparticle surface can dramatically change these barriers, triggering unwanted particle aggregations. It is thus crucial that the wrapping system be designed in a way as to maintain barriers that prevent aggregation. It is also important that the ‘wrapping agent’ does not complex multiple nanoparticles, which may also lead to the formation of multiparticle aggregates, and that the wrapper does not promote nanoparticle flocculation. We chose to work with commercially-available silica nanoparticles whose surfaces were functionalised with complexes of Ni^2+^ ions and nitrilotriacetic acid (Ni-NTA) ligands (Ni@SiNPs). Ni-NTA complexes are strong receptors for nitrogen-containing ligands, most notably the imidazole moieties of polyhistidine tags of recombinant proteins. Imidazole is known to be a good ligand for Ni^2+^ on account of it being a strong σ-donor, capability for π-backbonding, and providing further stabilisation through internal H-bonding within complexes.^25–27^ Additionally, at pH 4.5 imidazole ligands may be protonated, therefore alongside specific metal–ligand binding there would likely also be present a degree of electrostatic adsorption. We hypothesized that a SCPN decorated with a multivalent display of imidazole groups would possess a high degree of binding to Ni@SiNPs, the first step in the wrapping process.

### Preparation of polymer ‘wrapping agent’

**Fig. 2:**
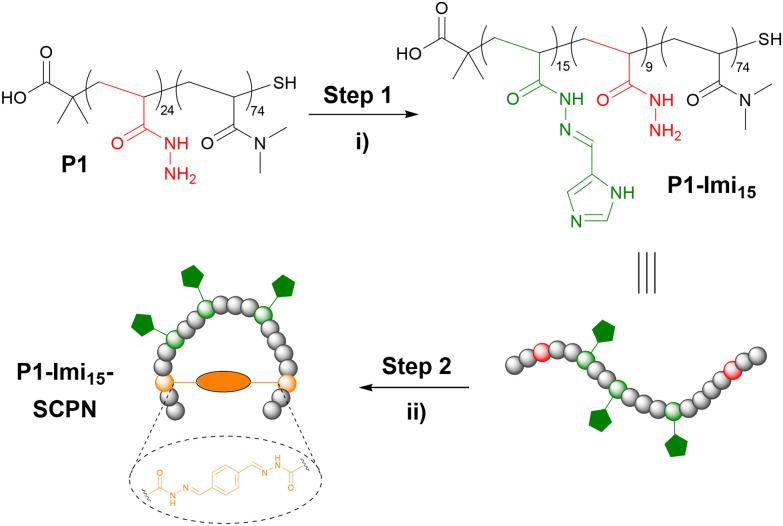
The synthesis of single-chain polymer nanoparticles (SCPNs) ‘wrapping agents’. (i) Post-polymerisation functionalisation of P1 with imidazole (4-imidazolecarboxaldehyde, 0.1 M AcOH/NH_4_OAc, pH 4.5, 18 h) to form the imidazole-decorated polymer P1-Imi_15_. (ii) Intramolecular crosslinking (terephthalaldehyde, 0.1 M AcOH/NH_4_OAc, pH 4.5, 18 h) to afford the ‘wrapping agent’ P1-Imi_15_-SCPN.

We designed a SCPN ‘wrapping agent’ based upon copolymer scaffold P1 (Fig. 2). The pendant hydrazide groups function as anchor points for the introduction of imidazole appendages and crosslinkers. P1 was prepared by the copolymerisation of Boc-acryloyl hydrazide^28^ (Fig. S1) with *N*,*N*-dimethylacrylamide (DMA) *via* radical addition–fragmentation chain-transfer (RAFT) polymerisation (Fig. S2) using optimised conditions,^29^ with subsequent Boc-deprotection (Fig. S3). DMA was incorporated into the scaffold to improve polymer solubility and aid characterisation. The degree of polymerisation (DP) and monomer composition were obtained *via* end-group analysis of the ^1^H NMR spectrum, and Boc-removal was confirmed through analysis by both ^1^H NMR spectroscopy and gel permeation chromatography (GPC; Fig. S5 and S6).

**Fig. 3:**
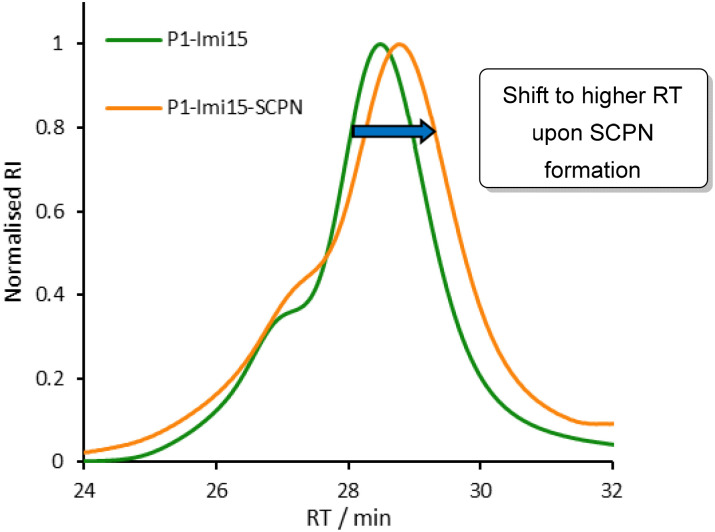
Overlaid normalised GPC chromatographic traces for P1-Imi_15_ (green) and P1-Imi_15_-SCPN (orange) highlighting the increase in peak retention time caused by intramolecular crosslinking; 1 g L^−1^ LiBr in DMF as eluent, calibrated with PMMA standards.

Post-polymerisation functionalisation was employed to introduce a multivalent display of ligands along the polymer scaffold. 4-Imidazole carboxaldehyde was conjugated to P1*via* acylhydrazone formation (Fig. 2, step 1) in 0.1 M AcOH/NH_4_OAc, pH 4.5 buffer solution, with 4-methoxyphenol included as a non-reactive internal standard to enable accurate determination of imidazole loading. ^1^H NMR spectroscopic analysis (Fig. S4) revealed that each polymer chain incorporated approximately 15 imidazole moieties (P1-Imi_15_); despite the use of excess aldehyde, this corresponds to functionalisation of 63% of the available hydrazide groups, consistent with previous reports.^30^ The crude product was purified by dialysis against water to remove unreacted aldehyde, and subsequent ^1^H NMR analysis confirmed that no hydrolysis of the acylhydrazone linkages had occurred. P1-Imi_15_ was then intramolecularly crosslinked into P1-Imi_15_-SCPN (Fig. 2, step 2) through the addition of a single equivalent of terephthalaldehyde to a dilute (10 μM) solution of P1-Imi_15_ in 0.1 M AcOH/NH_4_OAc, pH 4.5 buffer solution. Terephthalaldehyde was chosen as it forms hydrolytically stable aromatic acylhydrazones crosslinks that optimally undergo component exchange processes at pH 4.5, facilitating the structural metamorphosis process. Dialysis against pure water raises the solution pH to neutral, suppressing the dynamic behaviour of the acylhydrazone bonds and thereby preventing unwanted intermolecular crosslinking during concentration for characterisation. GPC analysis (Fig. 3) reveals the retention time (RT) has shifted to a larger value in comparison to P1-Imi_15_, a signature feature of intramolecular chain collapse^31–34^ that indicates the presence of a species with a smaller hydrodynamic radius and confirms the formation of P1-Imi_15_-SCPN. The presence of the shoulders on the peaks in the chromatograms are simply artifacts of the polymerisation and are hypothesised to arise on account of chain–chain terminations. ^1^H NMR spectroscopic and GPC data for all polymers (Table 1) highlights that each polymer was obtained with relatively low polydispersity index (PDI) values, suggesting a good level of control during polymerisation.

**Table 1:** NMR and GPC characterisation data of polymers

Polymer	*M*_n_ a (kDa)	PDI^b^	RT^b^ (min)	ΔRT^b^ (s)
P1	9.8	1.13	28.64	29.8
P1-Imi_15_	10.9	1.14	28.48	−9.6
P1-Imi_15_-SCPN	11.0	1.21	28.77	17.2

a Molecular weight calculated from ^1^H NMR spectra based on end group analysis.

b Data from GPC; 1 g L^−1^ LiBr in DMF as eluent, calibrated with PMMA standard.

### Optimisation of Ni@SiNPs colloidal stability

We envisaged that dynamic light scattering (DLS) could be used to monitor changes in particle diameter associated with binding of SCPNs onto their surfaces. However, to obtain unambiguous DLS measurements, the nanoparticles must exhibit good colloidal stability, ensuring that any increase in apparent particle size upon polymer addition can be attributed to formation of a surface-bound polymer layer rather than on account of particle aggregation. Thus, before conducting wrapping experiments the colloidal stability of the Ni@SiNPs was probed *via* measurements of zeta potential (ZP) and hydrodynamic radius (*D*_h_). Nanoparticles were considered stable if *D*_h_ values did not significantly increase over time and if the magnitude of their ZP was greater than 30 mV. The dynamic exchange of acylhydrazone bonds required for the structural metamorphosis necessitates that the wrapping process be conducted at pH 4.5. Exploration of a variety of aqueous buffers (Fig. S7) identified 10 mM AcOH/NH_4_OAc containing 0.1%_v/v_ Triton X-100 at pH 4.5 as a good dispersant because it was able to maintain a good level of colloidal stability throughout the time scale of the wrapping process. Decreasing the pH was found to decrease the magnitude of ZP to −14.2 mV (*cf.* −42.0 mV in pure water), probably on account of protonation of the silica surface. Despite this reduction in electrostatic repulsion, particle size measurements indicated maintenance of Ni@SiNPs colloidal stability (*D*_h_ = 276.7 ± 0.1 nm and PDI = 0.043), likely on account of steric stabilisation provided by the Triton X-100 surfactant. This buffer was used throughout the rest of this study (and is referred to simply as “the buffer solution”).

### Wrapping of Ni@SiNPs in crosslinked polymer films *via* structural metamorphosis

**Fig. 4:**
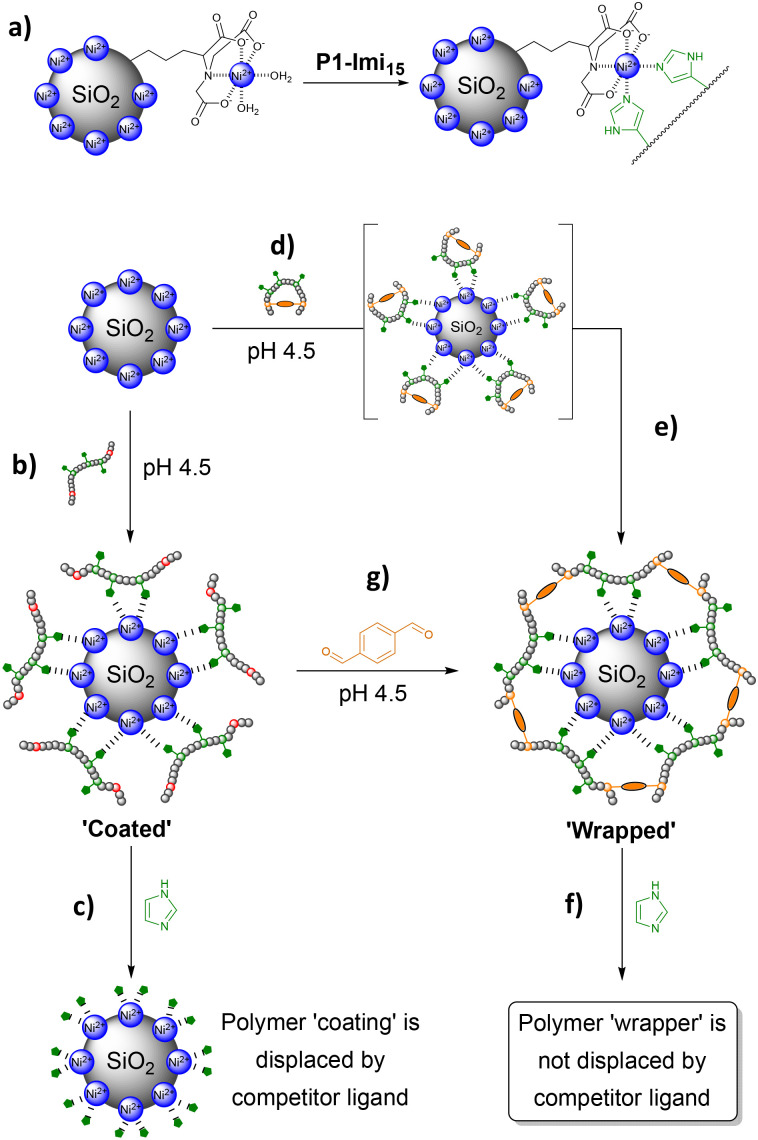
The ‘wrapping’ and ‘coating’ of Ni@SiNPs. (a) Bidentate binding of imidazole decorated polymers to Ni@SiNPs. (b) Incubation of Ni@SiNPs with P1-Imi_15_ results in the formation of a polymeric coating which (c) is easily displaced with imidazole. (d) Incubation of Ni@SiNPs with P1-Imi_15_-SCPN results in the adsorption of SCPNs upon the particle surface which then (e) reorganise their connectivity due to the concentration-triggered structural metamorphosis to wrap the nanoparticle in a polymer film which (f) cannot be displaced. (g) Crosslinker can be added to the ‘coated’ nanoparticles as an alternative route to the ‘wrapped’ nanoparticles.

**Fig. 5:**
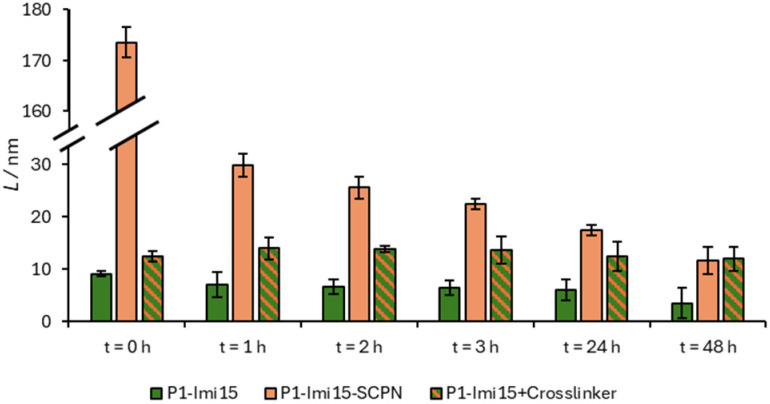
Polymer layer thickness, *L*, for Ni@SiNPs incubated with P1-Imi_15_ (green), P1-Imi_15_-SCPN (orange), or P1-Imi_15_**+**Crosslinker (green and orange striped) before and at multiple time (*t*) points after the addition of imidazole. Error bars indicate the standard error of the difference.

Ni@SiNPs (0.5 mg mL^−1^) were incubated with P1-Imi_15_-SCPN (Fig. 4d and e) in the buffer solution for 2 h with shaking and particle size was then obtained by DLS. Polymer layer thickness, *L*, was derived by subtracting the ‘naked’ particle size (276.7 nm) from the measured *D*_h_ and dividing by two. Particle size was found to increase to *D*_h_ ∼600 nm (PDI = 0.390), corresponding to *L* ∼170 nm (Fig. 5, *t* = 0 h). This initial large increase in *D*_h_ reflects transient aggregation rather than uncontrolled film growth, which we discuss later. An aliquot of a concentrated imidazole stock solution was then added to afford an imidazole concentration of 0.1 M, and the suspension was shaken for a further 1 h. A large decrease in *L* (∼170 nm → ∼30 nm) (Fig. 5, *t* = 1 h) was observed with a reduction in PDI from 0.378 to 0.115. The *D*_h_ measured for this suspension was then observed to continue to decrease over time with a trend fitting closely to a logarithmic decay (Fig. S8), before plateauing around 300 nm after 48 h, corresponding to a value of *L* = 11.7 nm (Fig. 5, *t* = 48 h, PDI = 0.083). In summary, the measured size of the species, which when taken together with the low PDI, indicates the formation of monodisperse individually wrapped nanoparticles.

The addition of excess imidazole should competitively displace polymer-conjugated imidazoles from the Ni@SiNPs. If the polymer chains have not extensively crosslinked with one another then the polymer should be displaced from the surface (Fig. 4c) with an accompanying decrease in particle size towards that of Ni@SiNPs. Conversely, if the polymer chains around the particles are extensively crosslinked, the competitor ligand would be unable displace the polymer layer (Fig. 4f) and a particle size consistent with the presence of a polymer layer would be retained. The observation that after addition of excess imidazole the layer thickness (*L* = 11.7 nm) remains larger than that of Ni@SiNPs supports the conclusion that structural metamorphosis of the bound polymer chains has occurred, yielding a reasonably monomodal population of Ni@SiNPs wrapped within a thin polymer film. The persistence of this particle size increase indicates that the polymer layer is not displaced and is therefore crosslinked. Moreover, the thickness of the polymer shell is comparable to the size of individual polymer chains measured in solution (∼6.5 nm, Fig. S9), consistent with the formation of a single, crosslinked polymer layer that effectively ‘wraps’ the nanoparticles.

This conclusion was supported by a control experiment in which Ni@SiNPs were incubated with P1-Imi_15_ (Fig. 4b). It was anticipated that a polymer layer should form upon the particle surface, however, it should be feasible to displace this layer upon addition of the competitor ligand. In this case, the particle is merely ‘coated’ rather than ‘wrapped’ within a cross-linked polymer film. Following incubation of the particles with the polymer, the value of *L* obtained was 9.1 nm (PDI = 0.058), which is consistent with a single polymer layer coating the particle. The very low PDI supports the conclusion of individually coated particles. Following treatment with imidazole the polymer layer was then observed to decrease in size from *L* = 9.1 nm to *L* = 3.5 nm after 48 h (Fig. 5, *t* = 48 h). At this point, the system had reached an equilibrium state with a small proportion of the polymer chains still bound to the surface. Although the particle size did not fully return to the value for Ni@SiNPs, the observation suggests that most of the surface-bound polymers were removed relatively easily on account of the lack of intermolecular crosslinks between the individual polymer chains.

**Fig. 6:**
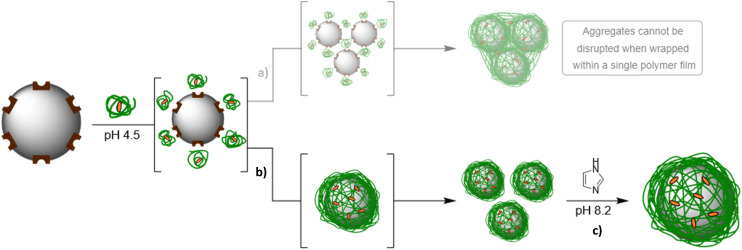
Representations of the hypothesised aggregation routes of Ni@SiNPs following P1-Imi_15_-SCPN addition. (a) Following adsorption of SCPNs to the particle it was hypothesised that aggregation of partially wrapped particles driven by the reduction in ZP upon polymer binding could occur. Structural metamorphosis of polymer chains that bind to different particles and that bind to multiple particles could then trap the aggregates within a covalently interconnected polymer film which would require chemical cleavage of covalent bonds to break apart the wrapped agglomeration. (b) Individual particles are completely wrapped following the binding of further SCPNs, triggering the concentration-driven structural metamorphosis. Aggregation of wrapped particles is driven by the reduction in ZP upon wrapping; however, disruption of aggregates can be triggered by the pH change associated with (c) imidazole addition to regain a monomodal dispersion of individually wrapped particles.

It was interesting to note that prior to treatment with imidazole, the layer thickness obtained with P1-Imi_15_-SCPN (*L* ∼170 nm) was much greater than that of an individually wrapped Ni@SiNPs (*L* = 11.7 nm). In fact, when left untreated, the recorded *D*_h_ for the particles in this suspension continued to increase over time and, when plotted alongside PDI, it was observed that both *D*_h_ and PDI increased concurrently (Fig. S10), suggesting that the particles were aggregating. Two possible routes that could be responsible for the observed aggregation were identified: multiple nanoparticles wrapped within a single polymer film (Fig. 6a) or polymer addition induced aggregation of individually wrapped particles (Fig. 6b). In the former case, addition of imidazole would not be expected to alter the measured size, as covalent intermolecular crosslinks between polymer chains cannot be disrupted by imidazole alone. This route can therefore be discarded as a possibility on account of the observed decrease in *D*_h_ upon addition of excess imidazole. Instead, these observations suggest that the addition of P1-Imi_15_-SCPN must have induced aggregation after individual particle wrapping had occurred.

This conclusion is reasonable, as the addition of any reagent to a nanoparticle suspension alters the steric and electronic environment at the particle surface and can therefore influence colloidal stability. The ZP of the individual polymer chains measured in the buffer solution was 0.3 ± 0.3 mV, indicating an effectively neutral surface charge. A positive ZP was expected as at pH 4.5 the imidazole groups on the polymer should be protonated, however a neutral ZP may be observed on account of trapping anions from the buffer solution. Consequently, if a particle becomes fully wrapped in a crosslinked film of these polymers, its apparent surface charge would also be expected to approach neutrality, at which point colloidal stability is diminished and aggregation may occur. The ZP of wrapped Ni@SiNPs aggregates was found to be slightly positive (6.3 ± 0.3 mV), but still sufficiently close to neutral to account for the observed aggregation.

These aggregates were readily disrupted upon addition of excess free imidazole. Imidazole is an amphoteric molecule with a basic p*K*_a_ of ∼7, and when added at concentrations substantially higher than those of the buffer components (0.1 M imidazole *versus* 10 mM buffer), it significantly increases the solution pH. Indeed, in the absence of nanoparticles, addition of imidazole in quantities comparable to those used in the wrapping experiments raised the buffer pH from 4.5 to 8.2, rendering the solution slightly basic. Under these conditions, the ZP of the individual polymer chains was measured as −25.0 ± 0.8 mV, compared to 0.3 ± 0.3 mV in the buffer solution. This shift to a negative ZP likely arises on account of deprotonation of the polymer imidazole groups. As expected, Ni@SiNPs wrapped in a polymer film at this higher pH similarly exhibited a strongly negative ZP (−20.8 ± 0.8 mV). The magnitude of this surface charge is sufficient to restore colloidal stability, resulting in the disruption of aggregates and redispersion of wrapped particles (Fig. 6c). Additionally, for Ni@SiNPs that are merely coated with P1-Imi_15_, the shift to negative polymer ZP upon imidazole addition generates an electrostatic repulsive force between the polymer and the particle which reinforces the displacement of non-intermolecularly crosslinked polymer chains. Therefore, imidazole was identified as playing multiple roles in this system: competitive ligand-exchange at Ni-NTA complexes, indirect modulation of colloidal stability through pH-induced changes to surface charge, and electrostatic displacement of discrete polymer chains.

To directly visualize this aggregation process and its subsequent disruption, transmission electron microscopy (TEM) was employed. Following deposition onto TEM grids, particle suspensions were stained with uranyl acetate contrast agent to enable visualisation of polymer material at the particle surface. Uranyl acetate staining produces a dark corona around the particle which makes the determination of the particle's edge difficult with the naked eye. Therefore, image analysis was performed by generating a plot profile to mathematically find the edges of the particle by identifying the darkest pixels in the image *i.e.* where the concentration of the stain was greatest. A random range of 20 particles were selected before and after wrapping so that a mean value and standard deviation could be calculated.

**Fig. 7:**
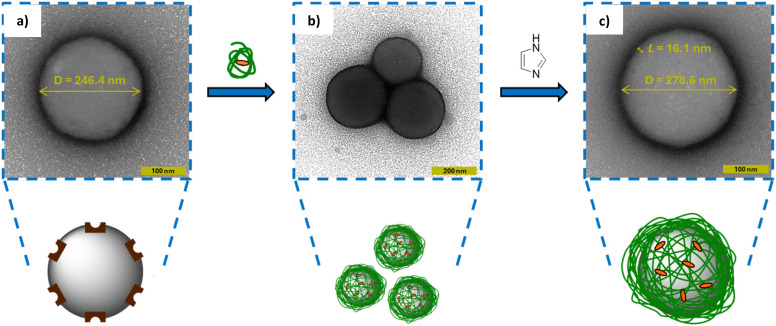
Wrapping affords aggregates of individually wrapped particles which can be disrupted through the addition of imidazole. TEM images of (a) Ni@SiNPs, (b) aggregates of individually wrapped particles prior to imidazole addition and (c) monodisperse, individually wrapped particles following imidazole addition. Following nanoparticle deposition, TEM grids were stained with uranyl acetate contrast agent to allow visualisation of the polymer layer. The images highlight both the increase in colloidal stability upon imidazole addition and the increase in particle size upon addition of the ‘wrapping agent’. Particle diameters (*D*) shown are mean values of a random range of 20 particles in each state and were obtained *via* image analysis relative to the scale bars indicated on each image. Below the TEM images are representations of the particles in each state.

Ni@SiNPs were first imaged (Fig. 7a) and exhibited a diameter of 246.4 ± 12.9 nm. As TEM measures the physical particle diameter (*D*) rather than the hydrodynamic diameter (*D*_h_), this value is expectedly smaller than the value obtained by DLS (276.7 nm). Following incubation with P1-Imi_15_-SCPN, multiparticle aggregates were observed (Fig. 7b), in agreement with DLS measurements. Upon treatment with imidazole, these aggregates were disrupted and a dispersion of discrete particles was recovered (Fig. 7c). The resulting particles displayed a larger size (*D* = 278.6 ± 9.7 nm) corresponding to a polymer layer thickness of ∼16 nm as determined by TEM, in close agreement with the value calculated from DLS (*L* = 11.7 nm). A *t*-test was performed on the two distributions which yielded a *p*-value ≪ 0.001 suggesting that they are statistically very different, which is highlighted by overlapping histograms overlaid with normal distribution curves (Fig. S12). Collectively, the TEM data corroborates the DLS-derived size analysis and provides direct visual confirmation of successful polymer wrapping at the nanoscale.

In contrast, large aggregates were not observed when Ni@SiNPs were incubated with non-crosslinked P1-Imi_15_ in buffer, despite this polymer exhibiting a similar ZP to the SCPN. We hypothesise that, in the absence of crosslinks, the resulting polymer layer is less dense and less effective at suppressing surface charge, allowing electrostatic repulsion between particles to maintain colloidal stability.

### Validation of the structural metamorphosis approach *via* an alternative wrapping route

An alternative route to wrap Ni@SiNPs was also explored which does not require the preparation of SCPNs. To the particles coated with P1-Imi_15_ in the buffer solution (*D*_h_ = 294.9 nm) was added a single equivalent of terephthalaldehyde (Fig. 4g) and the resulting suspension shaken for 1 h. The particle size (from DLS) increased slightly (*D*_h_ = 301.7 nm, Fig. 5, *t* = 0 h), suggesting that crosslinker addition promotes formation of a thicker polymer film, likely through sequestration of additional polymer chains from solution to complete surface wrapping. Addition of excess imidazole as a competitor ligand was observed to cause a small increase in particle size to 304.3 nm (Fig. 5, *t* = 1 h), and this value then decreased over time and plateaued around 300 nm (Fig. 5, *t* = 48 h). This observation supports the hypothesis that the crosslinker has been incorporated into the pre-existing polymer layer to form an intermolecularly crosslinked polymeric film, because, without the addition of the crosslinker, the P1-Imi_15_ layer was largely displaced after 48 h (*L* = 3.5 nm). Additionally, the final particle size observed was almost identical to that of the SCPN approach which reinforces the success of the structural metamorphosis and indicates that this is another viable method to wrap nanoparticles.

### Control experiments with non-complementary polymers revealed a degree of non-specific binding

To investigate the extent to which the wrapping process is driven by complementary interactions between polymer-conjugated imidazole ligands and Ni-NTA complexes on the nanoparticle surface, further control experiments were performed using a polymer lacking imidazole groups. A poly(*N*,*N*-dimethylacrylamide) homopolymer (PDMA) with DP = 99 as estimated by ^1^H NMR spectroscopy was prepared. This non-complementary polymer was chosen on account of structural similarities to P1-Imi_15_, which has DP = 98 and is ∼75% DMA by monomer composition.

Addition of PDMA to a suspension of Ni@SiNPs afforded *L* = 17.2 nm, which suggested polymer layer formation. However, this observation was accompanied by a concomitant increase in PDI, rising from 0.042 for Ni@SiNPs to 0.188 after PDMA incubation. The increase in PDI may indicate a degree of particle aggregation is responsible for the apparent increase in particle size rather than the formation of a uniform polymer layer, possibly arising from hydrophobic interactions between polymer chains associated with different particles. At pH 4.5 PDMA had a close to neutral ZP (−5.7 ± 0.2 mV) therefore hydrophobic interactions are likely significant in both polymer adsorption and aggregation. Alternatively, an increase in PDI could be observed with uncontrolled, non-specific adsorption of PDMA to the surface, leading to fluctuations in particle sizes and shapes.

Upon treatment with 0.1 M imidazole, the PDI of PDMA incubated Ni@SiNPs decreased from 0.188 to 0.105 after 24 h, which may suggest the disruption of aggregates as observed in the P1-Imi_15_-SCPN system. This shift towards a more monodisperse suspension may also reflect displacement of poorly-ordered, weakly-bound polymers from the particle surface. Although the calculated *L* value was only slightly smaller for PDMA than P1-Imi_15_ under identical conditions (*L* = 4.7 nm and 6.1 nm, respectively; Fig. S13), the decrease upon treatment with imidazole was far more substantial indicating that PDMA is more readily removed and therefore more weakly bound.

Despite this, a small residual PDMA layer remained, likely arising from non-specific interactions with the particle surface. These interactions would not be disrupted by the specific binding of imidazole to the Ni-NTA complex. At the slightly basic pH reached after imidazole addition, PDMA was found to maintain a close to neutral ZP (1.4 ± 0.6 MV) on account of the lack of ionisable groups present in the polymer structure, therefore there are no electrostatic repulsive forces to promote polymer displacement as with P1-Imi_15_.

Ultimately, while complementary polymer–particle interactions are central to an effective wrapping approach, within the P1-Imi_15_—Ni@SiNPs system a degree of non-specific binding is present. We speculate that replacement of the DMA comonomer for alternative, more hydrophilic comonomers may reduce non-specific interactions and further enhance wrapping fidelity.

## Conclusions

We have demonstrated that imidazole-decorated SCPNs can be used to successfully wrap Ni@SiNPs within thin, crosslinked polymer films. To the best of our knowledge, this represents the first application of the wrapping concept to nanoscale objects, a significant advance beyond our previous demonstrations on planar and microscale substrates.^12,13^ Central to this approach is a concentration-triggered structural metamorphosis, in which intramolecular crosslinks reorganise into intermolecular connections upon confinement at a nanoparticle surface. This process enables polymer film formation in a single step, without the need for sequential deposition or surface-initiated polymerisation.

The validity of this approach was reinforced through the demonstration of nanoparticle wrapping by an alternative, two-step pathway starting from non-crosslinked polymers, which ultimately led to the same wrapped end state. Successful wrapping was confirmed by the observation that, in the absence of crosslinks, imidazole-decorated polymers were displaced upon the addition of excess competitor ligand. Furthermore, control experiments using a non-complementary polymer revealed that, alongside metal–ligand binding, a degree of non-specific polymer–particle interactions also contribute to polymer layer formation.

More broadly, the successful wrapping of nanoscale objects within polymer films establishes a new design principle for nanoparticle surface modification that exploits polymer topology, dynamic covalent chemistry, and surface confinement to drive film formation. This concept may be extended to other systems involving alternative dynamic covalent bonds and polymer ligand–nanoscale receptor pairs, which could remove the pH 4.5 constraint (required for acylhydrazone exchange) and open the scope to a larger array of substrates. For example, this wrapping approach might be applied to complex and biologically relevant objects such as VLPs, where ligand-decorated SCPNs can be used to drive film formation through interactions with complementary surface proteins. Wrapping strategies of this kind may also prove useful for controlling colloidal stability, surface functionality, and release behaviour in nanoparticle-based delivery systems.^35,36^ Our approach would also benefit from the incorporation of a controlled release feature, where the polymer ‘wrapper’ can be removed upon the application of a trigger. Such a system would require the disruption of inter-chain connectivity, which we speculate could be achieved through incorporation of a functional group into the crosslinker which could be broken by orthogonal chemistry *e.g.* reductive cleavage of disulfide bonds. Overall, this study introduces a general and extensible framework for nanoparticle surface modification that leverages dynamic polymer architectures to access thin films in a controlled manner.

## Conflicts of interest

The authors declare no conflict of interest.

## Supplementary Material

PY-017-D6PY00112B-s001

## Data Availability

The data supporting this article have been included as part of the supplementary information (SI). Supplementary information: Fig. S1–S12 and further experimental details. See DOI: https://doi.org/10.1039/d6py00112b. Additional data are available upon request from the authors.
