# Supplementary material for: Dynamic covalent polymer films formed by structural metamorphosis at nanoparticle surfaces
Source: Polym Chem. 2026 Apr 20;17(19):1921–8. doi: 10.1039/d6py00112b (PMC13104708; doi:10.1039/d6py00112b)
Supplement: PY-017-D6PY00112B-s001 [file PY-017-D6PY00112B-s001.pdf]

Supplementary Information (SI) for Polymer Chemistry.
This journal is © The Royal Society of Chemistry 2026

Supporting Information

Dynamic Covalent Polymer Films Formed by Structural Metamorphosis at Nanoparticle Surfaces

Matthew J. Priestley and David. A. Fulton*

General Experimental

All chemicals were purchased from Sigma-Aldrich or Fisher Scientific and were used as received without further purification. Ni-NTA-functionalized silica nanoparticles (**Ni@SiNPs**) (sicastar®, 300 nm, Product # 43-48-302) were purchased from Micromod Partikeltechnologie GmbH (Germany).† All solvents were dried prior to use according to standard methods. *N,N*-dimethylacrylamide (**DMA**) was purified by vacuum distillation at 60 °C. All solvents used for flash chromatography were GPR grade. Unless state otherwise, all synthetic procedures were performed in oven-dried glassware under a $N_{2(g)}$ atmosphere.

Instrumentation

^{1}H and ^{13}C NMR spectra of synthesised compounds were recorded on a Bruker Avance 300 spectrometer (300 and 75 MHz respectively) or Bruker Avance 400 spectrometer (400 MHz and 100 MHz, respectively). In all cases, the residual solvent signal was used as an internal standard. Gel permeation chromatography (GPC) was conducted on a Malvern Omnisec instrument (Malvern Panalytcial) consisting of the Omnisec Resolve chromatography module and the Omnisec Reveal detector module equipped with a photodiode array detector with both ultraviolet and visible light sources, a multi-angle laser light scattering detector, refractive index detector and two PLgel 5 µm Mixed D 300 × 7.5 mm columns in series. Chromatogram analysis was performed on and analysed with the Omnisec software. Near monodisperse poly(methyl methacrylate) (PMMA) standards (Agilent Technologies) were used for calibration. Dynamic light scattering (DLS) measurements of particle size (D_h) and zeta potential (ZP) were performed on a Malvern Instruments Nano Series Zetasizer. Transmission electron microscopy (TEM) was performed using Hitachi HT7800 120kV TEM with cryo screening capabilities using an EMSIS CMOS Xarosa camera. pH measurements were made using a Hanna HI 90103 instrument which was calibrated before use using commercial buffer solutions (Sigma-Aldrich).

†Particle sizes for **Ni@SiNPs** measured by DLS (276.1 nm) and TEM (246.3 nm) were smaller than the reported value from the supplier (300 nm), however they were still within the desired nanoscale range and so were proceeded with for experiments.

N'-(*tert*-butoxycarbonyl)acryloylhydrazide (M1)

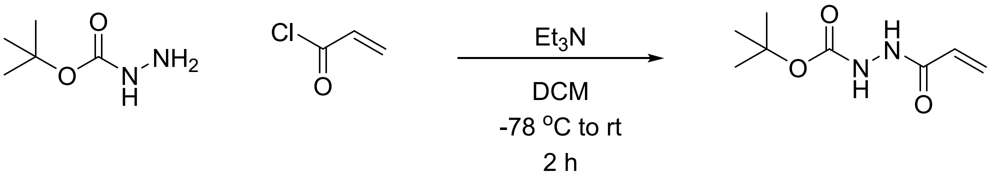

Figure S1A: Synthesis of Boc-protected acylhydrazide monomer **M1**.

Synthesis was adapted from a published procedure.[1] *Tert*-butyl carbazate (6.61 g, 50.0 mmol) was transferred to a round-bottomed flask with a stirrer bar and placed under a $N_{2(g)}$ atmosphere. Anhydrous DCM (100 mL) was added to dissolve. The mixture was cooled to -78 °C. EtN_3 (7.67 mL, 55 mmol) was added followed by acryloyl chloride (4.04 mL, 50 mmol) slowly. The mixture was stirred for 10 mins at -78 °C and then allowed to warm to room temperature with stirring for a further 2 h forming a white precipitate. H_2O (100 mL) was added to quench the reaction mixture. The aqueous layer was extracted with DCM (2 x 100 mL) and the combined organic phases were dried over $MgSO_4$, filtered and concentrated in vacuo to a yellow oil. The crude product was purified by flash column chromatography (6 x 20 cm SiO_2, EtOAc:hexane (1:4→2:3→3:2), R_f = 0.47 (3:2 EtOAc:hexane), $KMnO_4$ dip) to yield the title compound as a white solid (3.40 g, 18.3 mmol, 37%). ^{1}H NMR (400 MHz, $CDCl_3$) δ (ppm): 7.66 (br s, 1H, N*H*NHCOOC(CH_3)$_3$), 6.70 (br s, 1H, NHN*H*COOC(CH_3)$_3$), 6.41 (dd, 1H, C*H*=C), 6.15 (dd, 1H, C*H$_2$*=C), 5.76 (dd, 1H, C*H$_2$*=C), 1.48 (s, 9H, C(C*H$_3$*)$_3$).

Boc-Protected Acylhydrazide Polymer (Boc-P1)

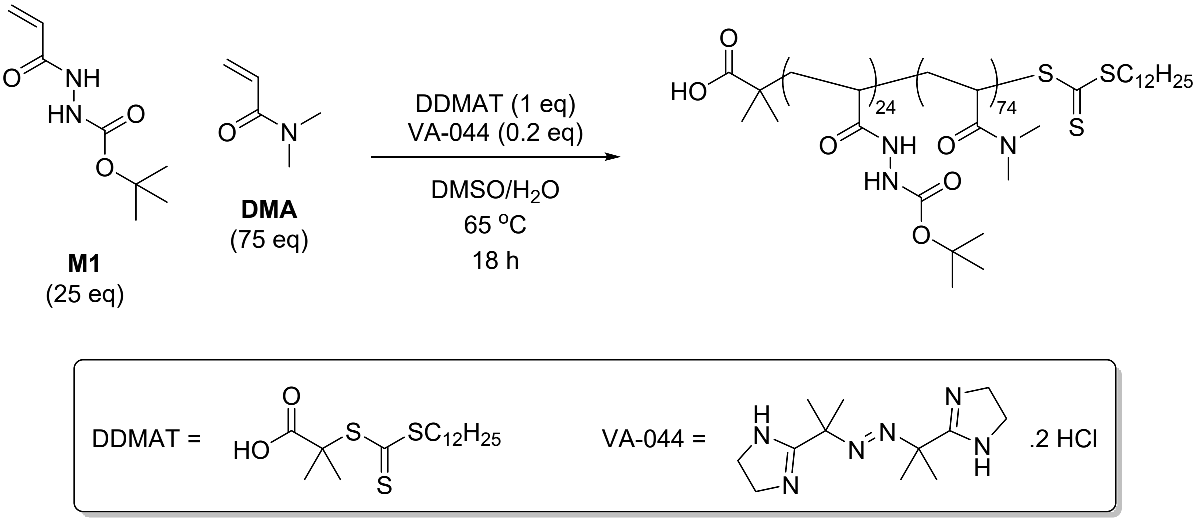

Figure S2A: Synthesis of Boc-protected polymer **P1** via the RAFT copolymerisation of **M1** and **DMA**. The structures of the chain transfer agent (DDMAT) and radical initiator (VA-044) are highlighted.

N'-(*tert*-butoxycarbonyl)acryloylhydrazide (**M1**) (25 eq, 250 mg, 1.34 mmol) and 2-(Dodecylthio-carbonothioylthio)-2-methylpropionic acid (DDMAT) (1 eq, 19.6 mg, 53.7 µmol) were added to a small Schlenk tube. DMSO (5 mL) was added to dissolve followed by *N,N'*-dimethylacrylamide (**DMA**) (75 eq, 0.415 mL, 4.03 mmol). 2,2'-Azobis[2-(2-imidazolin-2-yl)propane] dihydrochloride (VA-044) (0.2 eq, 3.47 mg, 10.7 µmol) was dissolved in H_2O and added to the Schlenk tube. The reaction mixture was degassed by five cycles of freeze-pump-thaw, then the vessel was backfilled and sealed under $N_{2(g)}$. The reaction mixture was then placed in a preheated oil bath at 65 °C with stirring for 18 h before quenching by opening the flask to the atmosphere. The reaction mixture was transferred into 3.5 kDa MWCO dialysis tubing and purified by dialysis against H_2O (3 x 2 L) over 36 h. The aqueous solution was lyophilised to obtain polymer **Boc-P1** as a white solid (612 mg, 50.3 µmol, 94 %). ^{1}H NMR (400 MHz, $CDCl_3$) δ (ppm): 8.0 – 10.0 (br, N*H*NHCOOC(CH_3)$_3$), 6.0 – 8.0 (br, NHN*H*COOC(CH_3)$_3$), 2.9 (br, N(CH_3)$_2$), 2.0 (br, C*H*CH_2, polymer backbone), 1.5 – 1.9 (br, CHC*H$_2$*, polymer backbone), 1.4 (br,

$C(CH_3)_3$, 0.9 (t, $(CH_2)_{11}CH_3$). The composition of **P1** was determined via ^1H NMR spectroscopic analysis prior to purification by dialysis by comparing the integration of the remaining, unreacted alkene protons with the $(CH_2)_{11}CH_3$ protons of DDMAT. This was reinforced by end-group analysis of the ^1H NMR spectrum of the pure polymer using the integration of the $N(CH_3)_2$ protons of **DMA** subunits and the $C(CH_3)_3$ protons of the **M1** subunits, again compared to the $(CH_2)_{11}CH_3$ protons of DDMAT.

Acylhydrazide Polymer (P1)

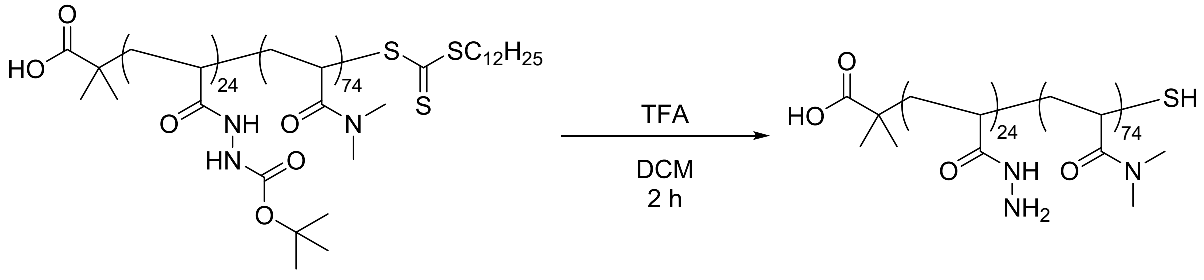

Figure S3A: Removal of the Boc-protecting group to give **P1**.

Boc-P1 (600 mg, 49.3 µmol) was dissolved in DCM (10 mL). Trifluoroacetic acid (TFA) (10 mL) was added, and the solution was stirred at room temperature for 2 h. The solution was concentrated in vacuo to a yellow oil which was redissolved in H_2O before purification by dialysis against H_2O (3 x 2 L) over 36 h. The aqueous solution was lyophilised to obtain polymer **P1** as a white solid (462 mg, 47.3 µmol, 96 %). ^1H NMR (400 MHz, D_2O) δ (ppm): 2.8 – 3.2 (br, $N(CH_3)_2$), 2.4 – 2.8 (br, C*H*CH_2, polymer backbone), 1.4 – 1.8 (br, CHCH_2, polymer backbone).

Imidazole-Conjugated Polymer (P1-Imi$_{15}$)

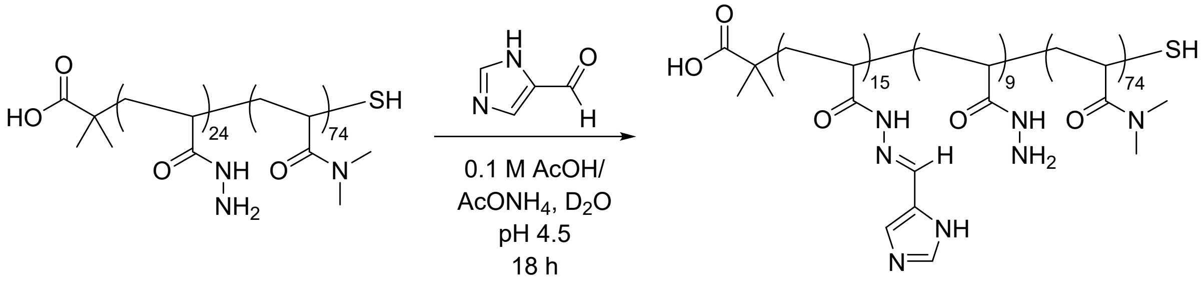

Figure S4A: Conjugation of 4-imidazole carboxaldehyde onto the **P1** scaffold via acylhydrazone formation.

P1 (34.0 mg, 3.48 µmol) transferred to a vial. 4-Imidazole carboxaldehyde (19.5 mg, 203 µmol) and 4-methoxy phenol (4.8 mg, 38.7 µmol) were transferred to a separate vial followed by addition of 0.1M AcOH/$AcONH_4$ D_2O pH 4.5 (1 mL) with sonication to dissolve. An aliquot (856 µL) of this solution was transferred into the vial containing **P1** so that there were 50 mol eq. of aldehyde compared to polymer (9.5 mol eq. 4-methoxy phenol). Stirred at room temperature for 18 h and then transferred to NMR tube for analysis. The reaction mixture was transferred into 3.5 kDa MWCO dialysis tubing and purified by dialysis against H_2O (3 x 0.5 L) over 36 h. The aqueous solution was lyophilised to obtain polymer **P1-Imi$_{15}$** as a white solid (30 mg, 2.74 µmol, 79 %). ^1H NMR (400 MHz, D_2O) δ (ppm): 7.1 – 8.3 (br, Ar*H*, C*H*=NNH), 2.8 – 3.2 (br, $N(CH_3)_2$), 2.4 – 2.8 (br, C*H*CH_2, polymer backbone), 1.2 – 2.0 (br, CHCH_2, polymer backbone). The degree of imidazole loading was calculated by ^1H NMR spectroscopic analysis prior to purification by dialysis. The remainder of the 4-Imidazole carboxaldehyde/4-methoxy phenol solution was diluted with D_2O and NMR analysis was performed to give the ratio of C_6H_4 signals to C*H*O (aldehyde)/C*H*$(OH)_2$ (hydrate) signals before conjugation. This ratio was compared to that in the ^1H NMR spectrum after the reaction and revealed that 15 aldehyde units had been conjugated onto the polymer scaffold.

Imidazole-Conjugated Single-Chain Polymer Nanoparticle (P1-Imi$_{15}$-SCPN)

P1-Imi$_{15}$ (13 mg, 1.19 µmol) was dissolved in 0.1M AcOH/$AcONH_4$ pH 4.5 (120 mL) so that [**P1-Imi$_{15}$**] = 10 µM. Terephthalaldehyde (275 µL, 4.32 mM in H_2O, 1.19 µmol) was added and the solution was stirred at room temperature for 18 h. The reaction mixture was transferred into 3.5 kDa MWCO dialysis tubing and purified by dialysis against H_2O (3 x 2 L)

over 36 h. The aqueous solution was lyophilised to obtain polymer **P1-Imi$_{15}$-SCPN** as a white solid (10 mg, 0.91 µmol, 77 %). GPC analysis confirmed successful SCPN formation.

Protocol for DLS Measurements of Ni@SiNPs

Ni@SiNPs (8.5 µL, 50 mg/mL in H_2O) were transferred to an Eppendorf. The buffer solution (841.5 µL) was added to give [**Ni@SiNPs**] = 0.5 mg/mL and the suspension was filtered through a 1.5 µm Nylon syringe filter. The suspension was transferred to a plastic UV cuvette for measurement of D_h and to a zeta potential cell for measurement of ZP.

General Protocol for DLS Wrapping Experiments

Ni@SiNPs (8.5 µL, 50 mg/mL in H_2O) were transferred to an Eppendorf. The buffer solution (741.5 µL) was added to give [**Ni@SiNPs**] = 0.567 mg/mL. Polymer (100 µL, 100 µM in the buffer solution) was added to give [**Ni@SiNPs**] = 0.5 mg/mL and [Polymer] = 11.7 µM and the suspension was filtered through a 1.5 µm Nylon syringe filter. The suspension shaken at room temperature for 2 h and then was transferred to a plastic UV cuvette for measurement of D_h and to a zeta potential cell for measurement of ZP. The suspension was returned to the Eppendorf and imidazole (29.3 µL, 3 M in H_2O) was added to give [imidazole] = 0.1 M. The suspension was shaken at room temperature and DLS measurements were made at several time points.

Protocol for DLS Wrapping Experiment via the Two-Step Pathway

Ni@SiNPs (8.5 µL, 50 mg/mL in H_2O) were transferred to an Eppendorf. The buffer solution (741.5 µL) was added to give [**Ni@SiNPs**] = 0.567 mg/mL. **P1-Imi$_{15}$** (100 µL, 100 µM in the buffer solution) was added to give [**Ni@SiNPs**] = 0.5 mg/mL and the suspension was filtered through a 1.5 µm Nylon syringe filter. The suspension shaken at room temperature for 2 h and then was transferred to a plastic UV cuvette for measurement of D_h and to a zeta potential cell for measurement of ZP. The suspension was returned to the Eppendorf and terephthalaldehyde (4.6 µL, 4.32 mM in H_2O) was added to give [terephthalaldehyde] = 2[**P1-Imi$_{15}$**]. The suspension was shaken at room temperature for 1 h and then was transferred to a plastic UV cuvette for measurement of D_h. The suspension was returned to the Eppendorf and imidazole (29.3 µL, 3 M in H_2O) was added to give [imidazole] = 0.1 M. The suspension was shaken at room temperature and DLS measurements were made at several time points.

References

1. T. Flagstad, M. R. Hansen, S. T. Le Quement, M. Givskov and T. E. Nielsen, *ACS Combinatorial Science*, 2015, **17**, 19-23.

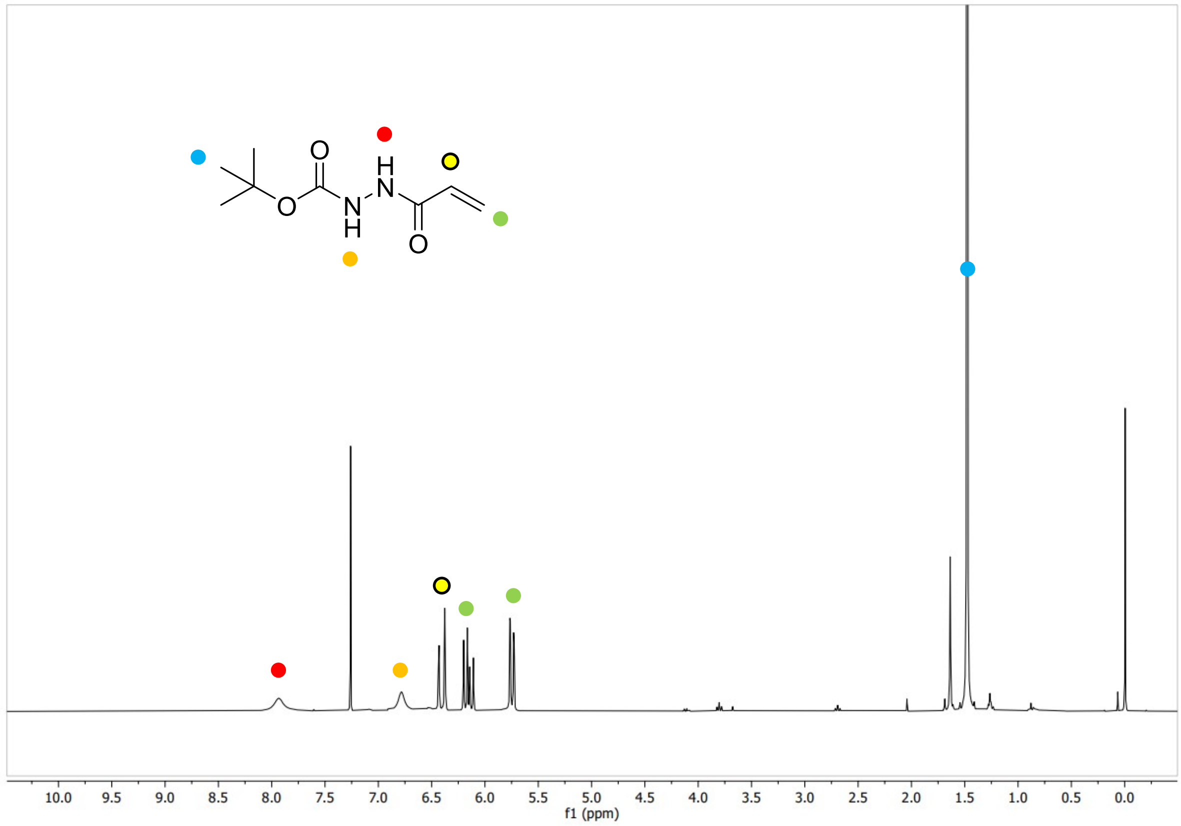

Figure S1B: ^{1}H NMR spectrum of **M1** with peak assignment.

Appendix

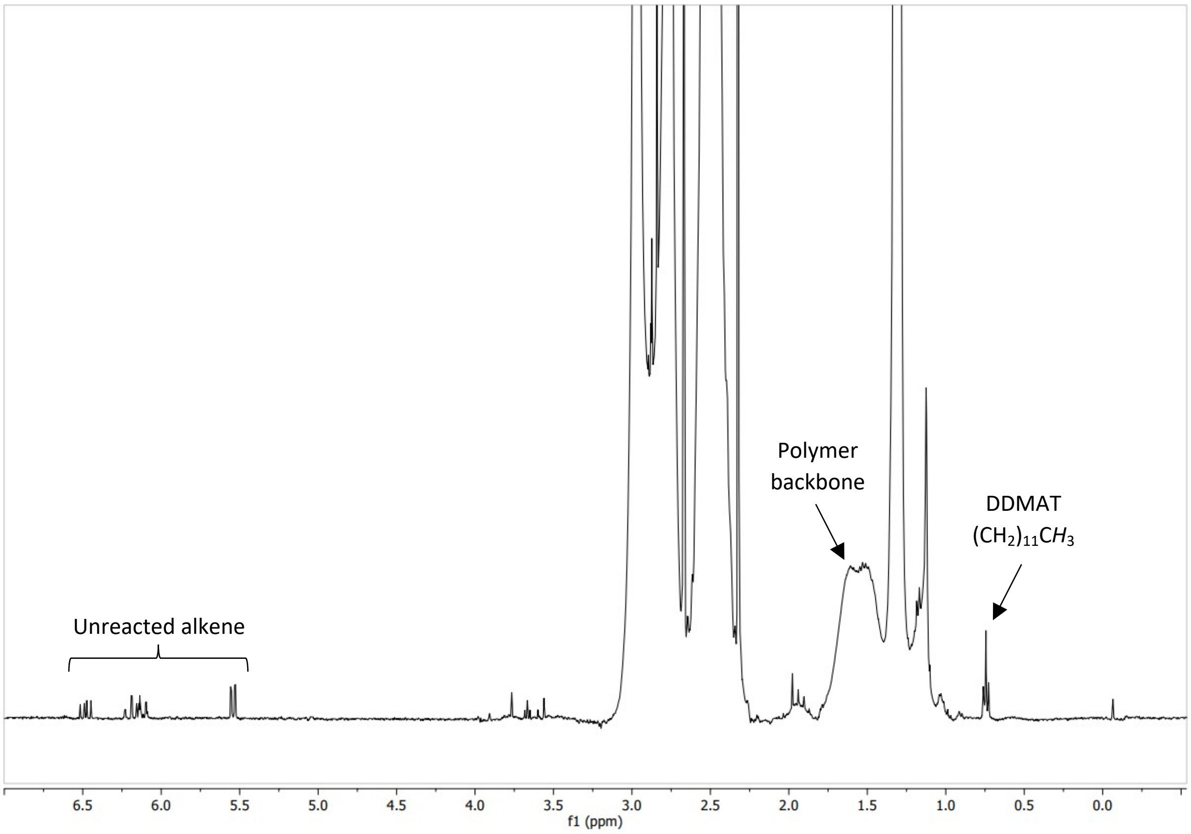

Figure S2B: ^{1}H NMR spectrum of crude **Boc-P1** prior to purification by dialysis. The unreacted alkene and DDMAT signals highted were used to calculate the degree of polymerisation. The presence of the broad polymer backbone peak is also highlighted.

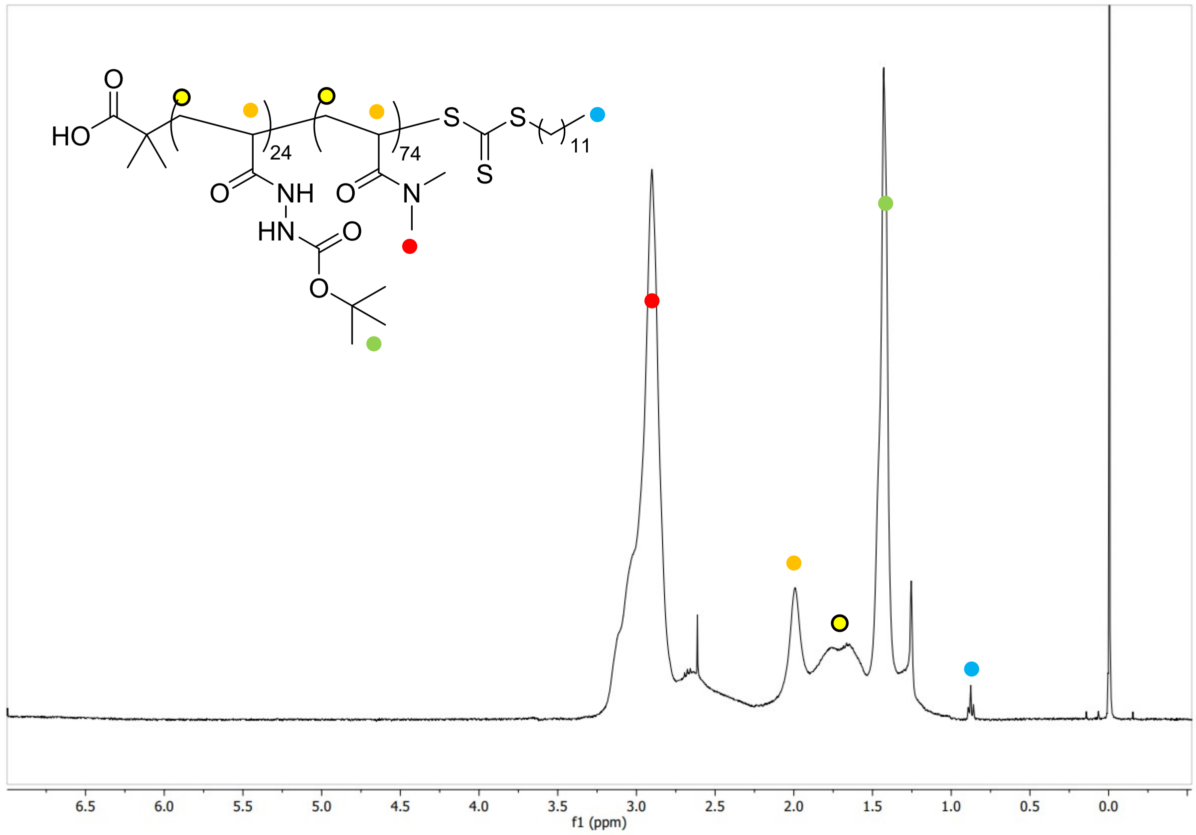

Figure S2C: ^{1}H NMR spectrum of pure **Boc-P1** after purification by dialysis with peak assignment.

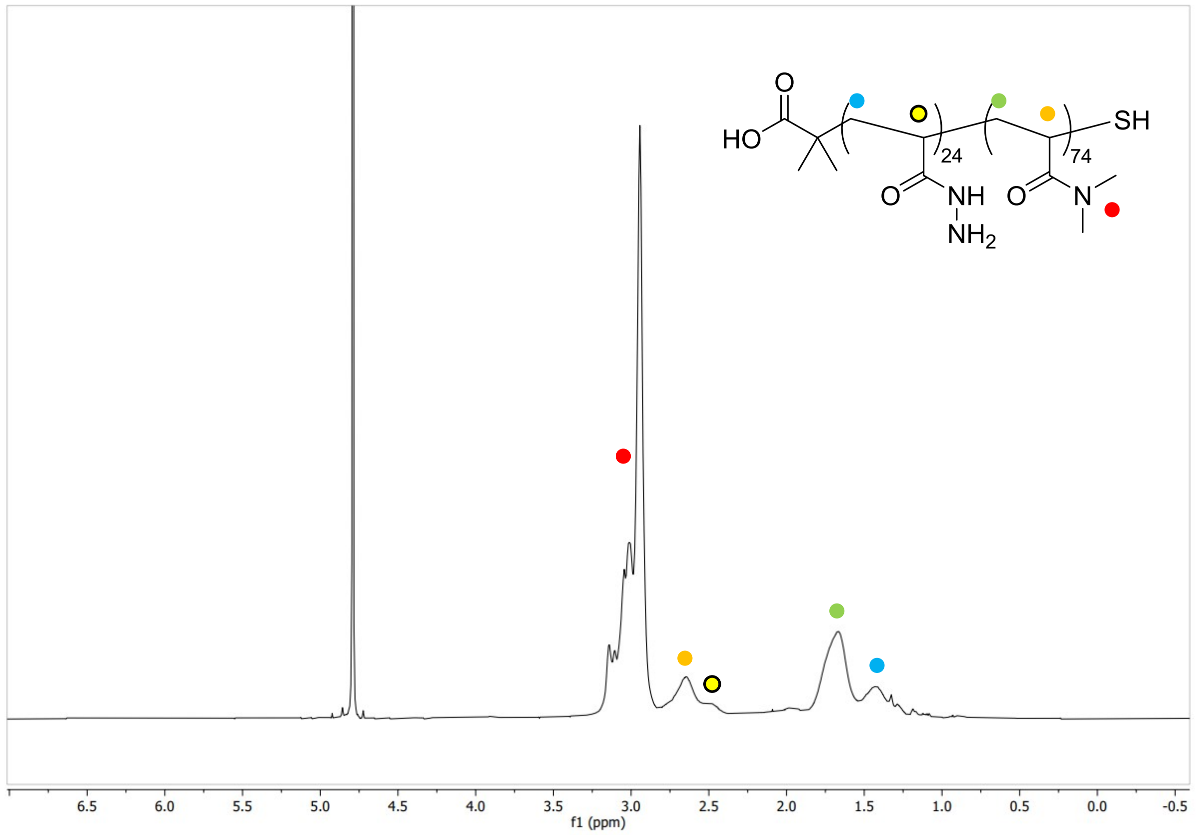

Figure S3B: ^{1}H NMR spectrum of pure **P1** after purification by dialysis with peak assignment.

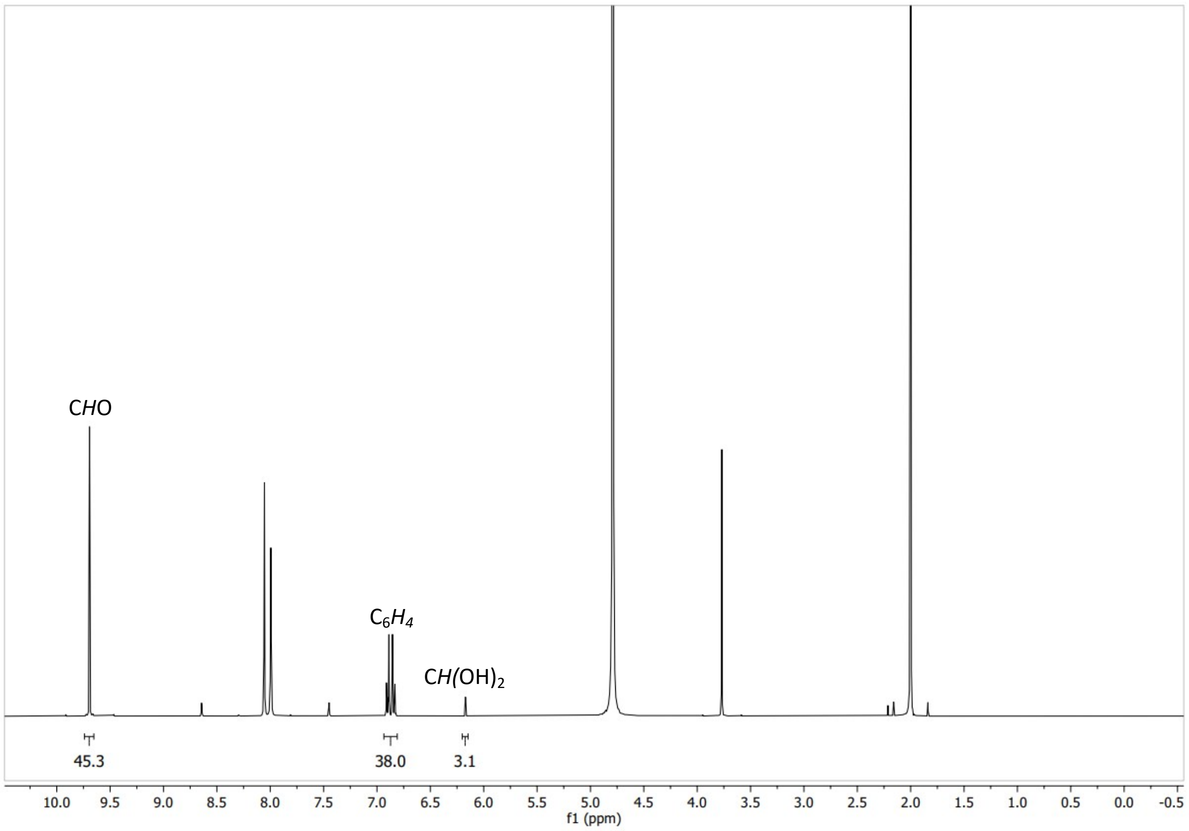

Figure 4B: ^{1}H NMR spectrum of 4-imidazole/4-methoxy phenol solution in 0.1 M AcOH/$AcONH_4$, D_2O, pH 4.5 prior to conjugation onto **P1**. The aromatic C_6H_4 signal of 4-methoxy phenol and the aldehyde C*H*O and hydrate C*H*$(OH)_2$ signals of 4-imidazole carboxaldehyde highlighted were used to calculate the imidazole loading onto the polymers.

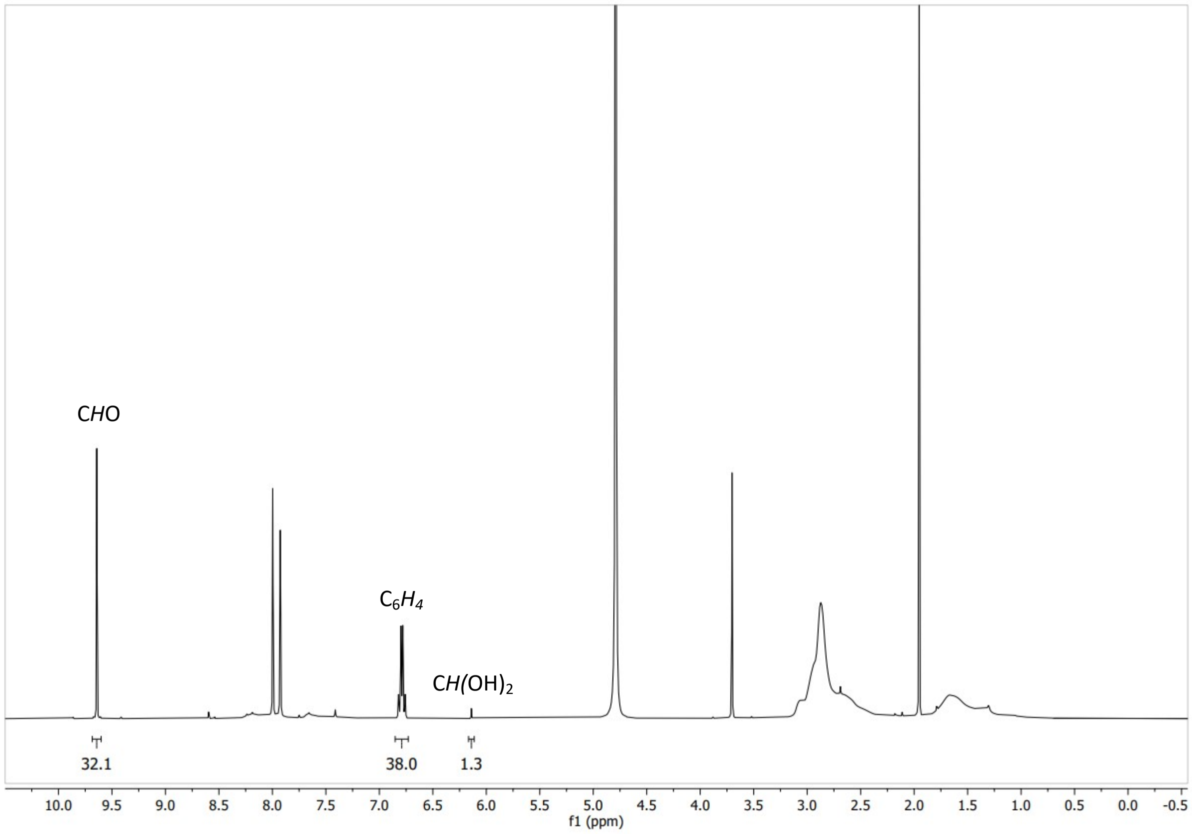

Figure S4C: ^{1}H NMR spectrum of reaction mixture for imidazole conjugation onto **P1** in 0.1 M AcOH/$AcONH_4$, D_2O, pH 4.5 prior to purification by dialysis. The aromatic C_6H_4 signal of 4-methoxy phenol and the aldehyde C*H*O and hydrate C*H*$(OH)_2$ signals of 4-imidazole carboxaldehyde highlighted were used to calculate the imidazole loading onto the polymers.

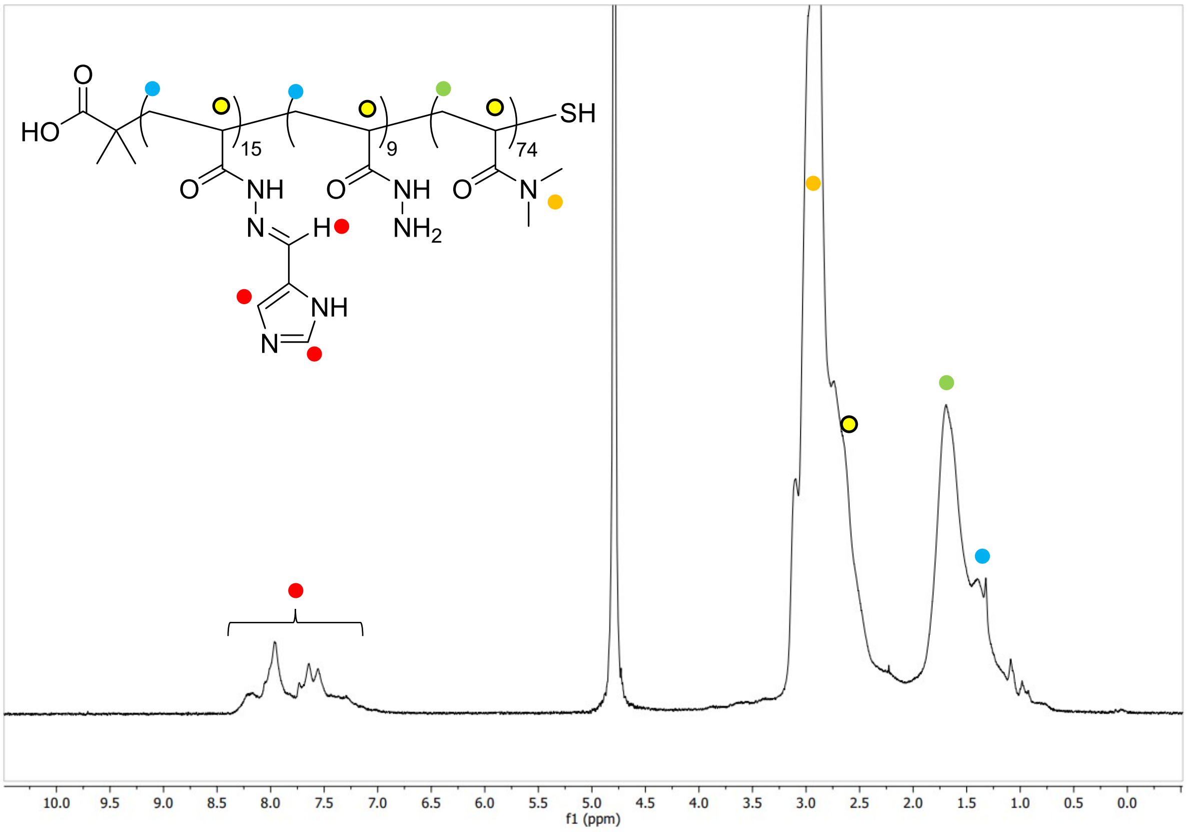

Figure S4D: ^{1}H NMR spectrum of pure **P1-Imi$_{15}$** after purification by dialysis with peak assignment.

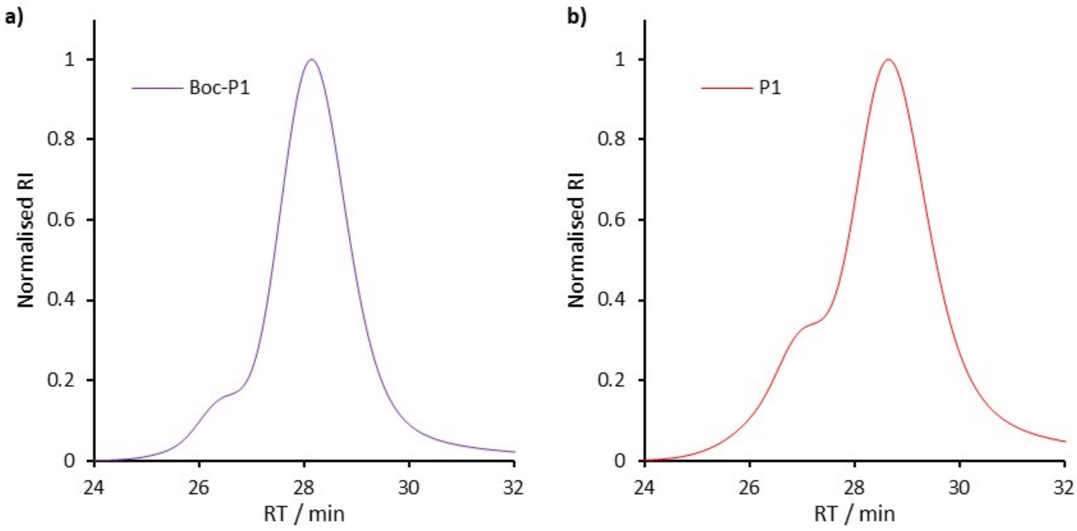

Figure S5: Normalised GPC chromatographic traces for a) **Boc-P1** and b) **P1**; 1 g/L LiBr in DMF as eluent, calibrated with PMMA standards.

Polymer	M_n[a] (kDa)	M_n[b] (kDa)	M_w[b] (kDa)	PDI[b]	RT[b] (min)	ΔRT[b] (s)
**Boc-P1**	12.2	18.6	20.3	1.09	28.15	-
**P1**	9.8	16.4	18.5	1.13	28.64	29.8
**P1-Imi$_{15}$**	10.9	17.1	19.4	1.14	28.48	-9.6
**P1-Imi$_{15}$-SCPN**	11.0	15.6	18.9	1.21	28.77	17.2

Figure S6: NMR and GPC characterisation data of polymers. [a]Molecular weight calculated from ^{1}H NMR spectra based on end group analysis. [b]Data from GPC; 1 g/L LiBr in DMF as eluent, calibrated with PMMA standard.

Dispersant	ZP / mV	SD / mV	Comment on D_h	Comment on pH
**H_2O**	-42.0	0.8	Stable	☒
**0.1%$_{v/v}$ Triton X-100**	-28.6	0.6	Stable	☒
**0.1 M NaCl**	-27.3	0.9	Stable	☒
**0.1%$_{v/v}$ Triton X-100<br>0.1 M NaCl**	-3.1	0.8	Aggregated	☒
**0.1 M AcOH/$AcONH_4$**	-25.0	2.6	Aggregated	☑
**0.1%$_{v/v}$ Triton X-100<br>0.1 M AcOH/$AcONH_4$**	-3.3	0.3	Aggregated	☑
**10 mM AcOH/$AcONH_4$**	-33.8	0.1	Aggregated	☑
**0.1%$_{v/v}$ Triton X-100<br>10 mM AcOH/$AcONH_4$**	-14.2	0.5	Stable	☑
**0.01%$_{v/v}$ Triton X-100<br>10 mM AcOH/$AcONH_4$**	-16.8	0.6	Aggregated	☑

Figure S7: Investigation of colloidal stability in of **Ni@SiNPs** in different dispersants by measurement of ZP and D_h. Entry 8 was used as the buffer solution for wrapping experiments as the D_h was stable and the pH allowed acylhydrazone exchange.

		D_h / nm	SD / nm	n	*L* / nm	SE_{Diff} / nm	PDI
t = 0 h	Control	276.7	0.1	3	0.0	0.1	0.043
	**P1-Imi$_{15}$**	294.9	1.2	6	9.1	0.5	0.058
	**P1-Imi$_{15}$-SCPN**	623.9	6.1	4	173.6	3.1	0.390
	**P1-Imi$_{15}$+Crosslinker**	301.7	2.3	5	12.5	1.0	0.074
t = 1 h	**P1-Imi$_{15}$**	290.9	4.1	3	7.1	2.4	0.054
	**P1-Imi$_{15}$-SCPN**	336.3	3.7	3	29.8	2.2	0.115
	**P1-Imi$_{15}$+Crosslinker**	304.6	3.4	3	14.0	2.0	0.057
t = 2 h	**P1-Imi$_{15}$**	290.0	3.3	5	6.7	1.5	0.049
	**P1-Imi$_{15}$-SCPN**	327.8	5.2	6	25.6	2.1	0.129
	**P1-Imi$_{15}$+Crosslinker**	304.3	1.2	5	13.8	0.5	0.054
t = 3 h	**P1-Imi$_{15}$**	289.5	3	5	6.4	1.3	0.068
	**P1-Imi$_{15}$-SCPN**	321.5	1.7	3	22.4	1.0	0.137
	**P1-Imi$_{15}$+Crosslinker**	304.0	4.4	3	13.7	2.5	0.048
t = 24 h	**P1-Imi$_{15}$**	288.8	4.5	5	6.1	2.0	0.040
	**P1-Imi$_{15}$-SCPN**	311.6	1.8	3	17.5	1.0	0.089
	**P1-Imi$_{15}$+Crosslinker**	301.4	2.1	4	12.4	1.2	0.086
t = 48 h	**P1-Imi$_{15}$**	283.7	4.9	3	3.5	2.9	0.043
	**P1-Imi$_{15}$-SCPN**	300.0	6.4	6	11.7	2.7	0.083
	**P1-Imi$_{15}$+Crosslinker**	300.6	5.7	6	12.0	2.4	0.093

Figure S8: DLS measurements (D_h and PDI) for the data presented in Figure 5. The number of measurements (n) over which the standard deviation (SD) of each entry was calculated is shown. The polymer layer thickness (*L*) was calculated by finding the difference between the D_h of each measurement and that of the control, then dividing by two. The standard error of the difference (SE_{Diff}) was calculated to find the error in the calculated of *L*.

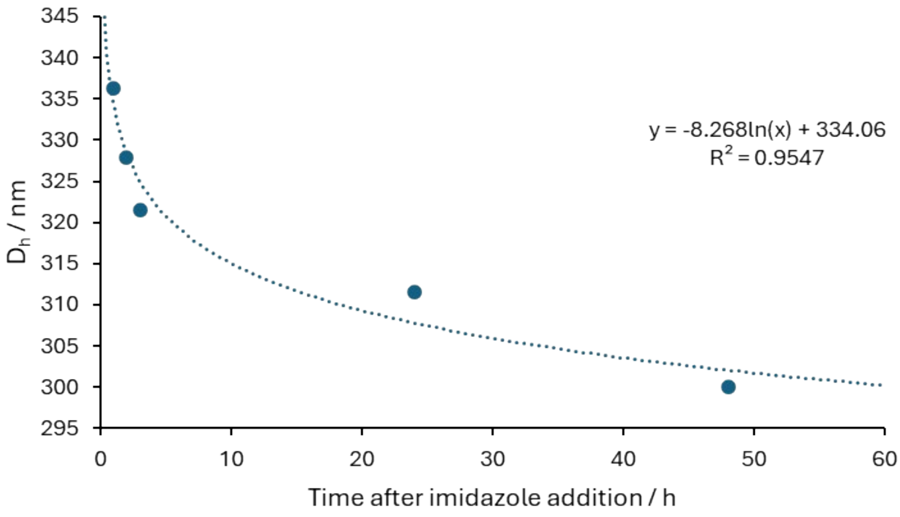

Figure S8: Plot of DLS-derived D_h against time for **Ni@SiNPs** incubated with **P1-Imi$_{15}$-SCPN** following treatment with 0.1 M imidazole. The data fits very closely to the logarithmic trendline shown.

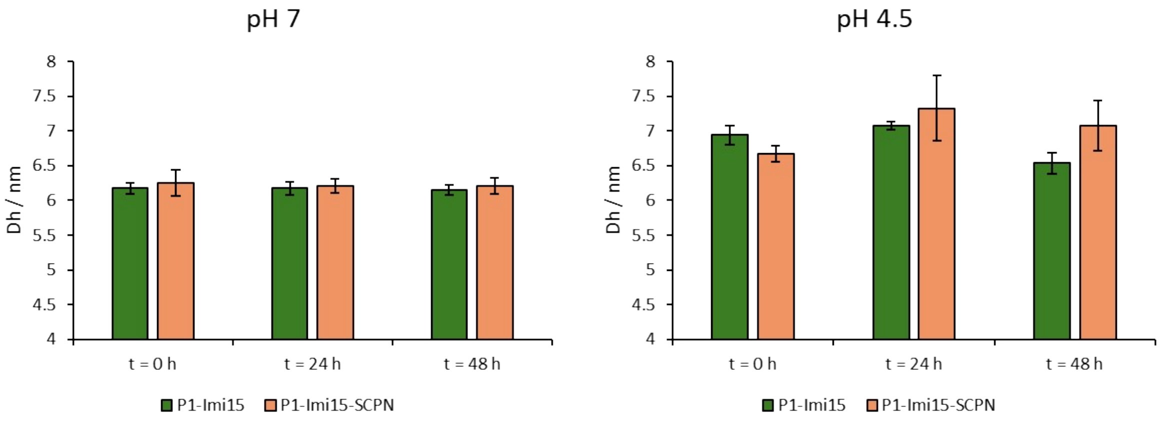

Figure S9: Plot of DLS-derived D_h against time for **P1-Imi$_{15}$** and **P1-Imi$_{15}$-SCPN** in both H_2O (pH 7) and the buffer solution (pH 4.5) used in the wrapping experiments. Polymer solutions were prepared at the same concentration used in the wrapping experiments (11.7 μM).

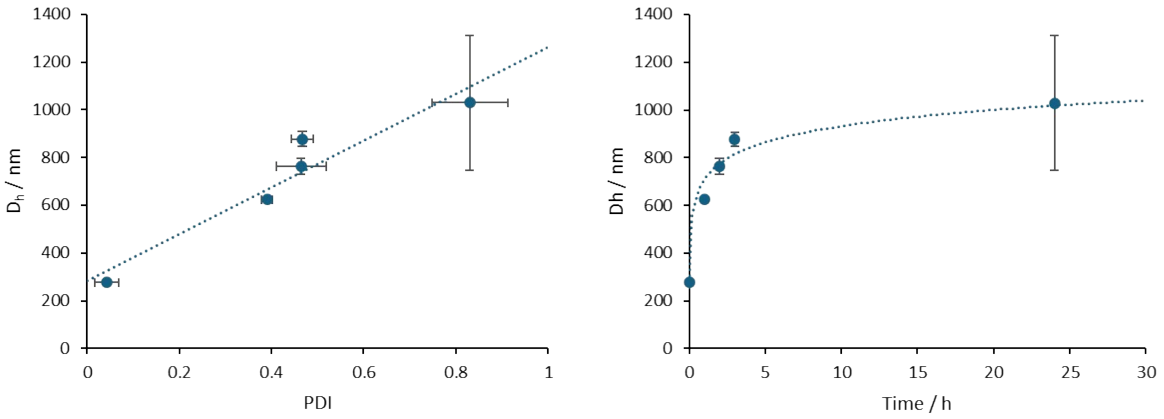

Figure S10: Plot of DLS-derived D_h against PDI (left) and time (right) for **Ni@SiNPs** incubated with **P1-Imi$_{15}$-SCPN** that were not treated with imidazole. The linear relationship between D_h and PDI suggests aggregation. The increase in D_h slows over time suggesting that an equilibrium state is achieved.

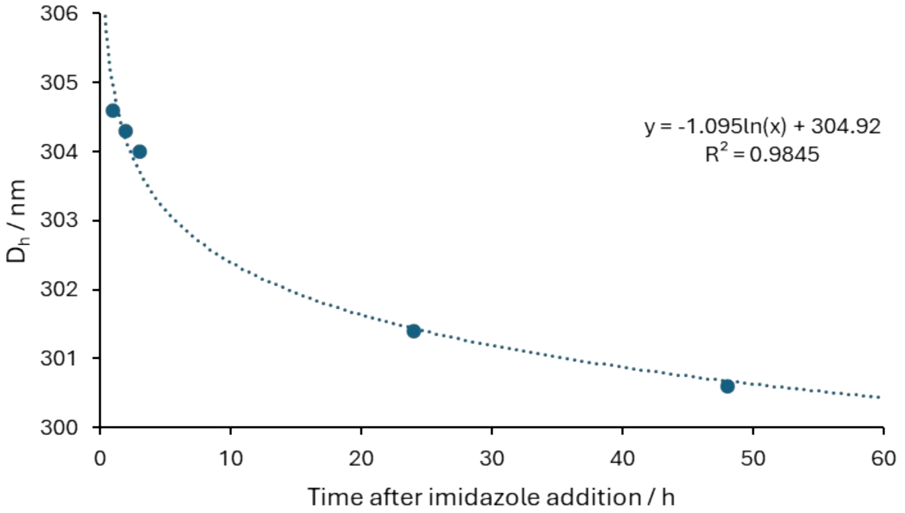

Figure S11: Plot of DLS-derived D_h against time for **Ni@SiNPs** incubated with **P1-Imi$_{15}$+Crosslinker** following treatment with 0.1 M imidazole. The data fits very closely to the logarithmic trendline shown.

Diameter / nm	Control	Wrapped
1	261.441	281.802
2	261.081	283.964
3	250.63	289.369
4	238.018	274.774
5	231.172	284.505
6	234.415	266.127
7	253.694	262.523
8	246.847	286.487
9	268.288	280.36
10	261.261	279.279
11	235.316	272.432
12	239.099	259.82
13	218.919	272.252
14	255.495	290.45
15	247.027	270.811
16	250.45	284.504
17	260	267.747
18	246.306	283.243
19	235.855	286.486
20	233.333	294.234

	Mean Diameter / nm	Standard Deviation / nm	Polymer layer thickness / nm	Standard Error of the Difference / nm	*t*-statistic	*p*-value
Control	246.4	12.9	16.1	1.8	8.9	1.5×10^{-10}
Wrapped	278.6	9.7				

Figure S12A: Raw size data for the random selection of 20 particles from the TEM grids of **Ni@SiNPs** before (Control) and after (Wrapped) the particles were wrapped within an interconnected polymer film and the subsequent statistical analysis to obtain the *p*-value.

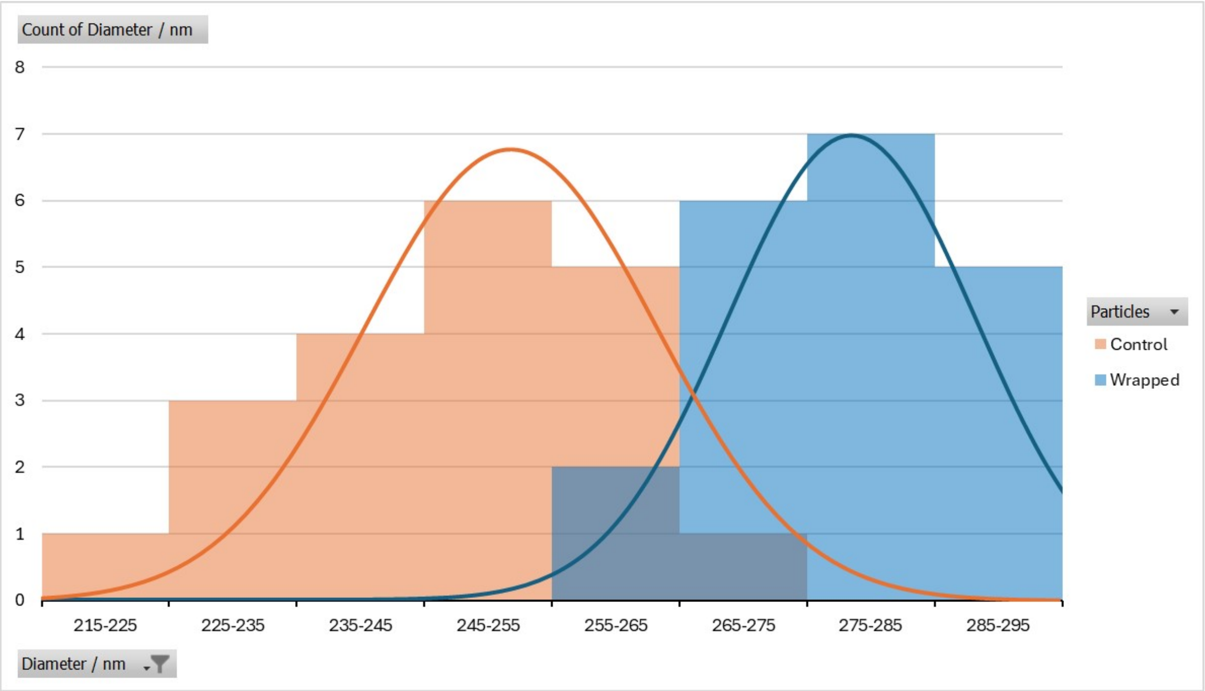

Figure S12B: Overlapping histograms and normal distribution curves for the random selection of 20 particles from the TEM grids of **Ni@SiNPs** before (Control, orange) and after (Wrapped, blue) the particles were wrapped within an interconnected polymer film.

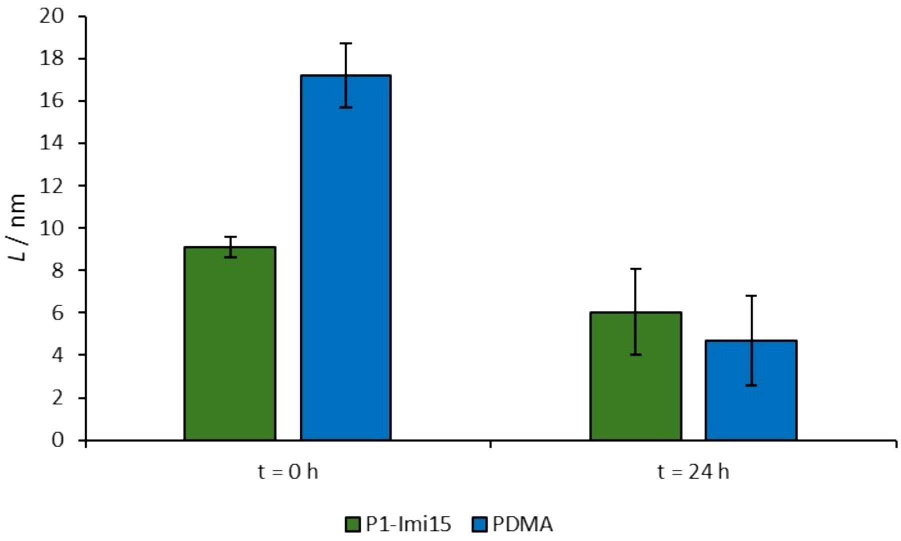

Figure S13: Polymer layer thickness, *L*, for **Ni@SiNPs** incubated with **P1-Imi$_{15}$** (green) or **PDMA** (blue) before (t = 0 h) and after (t = 24 h) the addition of imidazole. Error bars indicate the standard error of the difference.
